# Antioxidant Activity of Leaf Extracts from *Stevia rebaudiana* Bertoni Exerts Attenuating Effect on Diseased Experimental Rats: A Systematic Review and Meta-Analysis

**DOI:** 10.3390/nu15153325

**Published:** 2023-07-26

**Authors:** Maria Papaefthimiou, Panagiota I. Kontou, Pantelis G. Bagos, Georgia G. Braliou

**Affiliations:** 1 Department of Computer Science and Biomedical Informatics, University of Thessaly, 35 131 Lamia, Greece; mpapaefthymiou@uth.gr (M.P.); pbagos@compgen.org (P.G.B.); 2 Department of Mathematics, University of Thessaly, 35 132 Lamia, Greece; pkontou@compgen.org

**Keywords:** stevia, antioxidant, meta-analysis, animal model

## Abstract

Stevia (*Stevia rebaudiana* Bertoni) is an aromatic plant known for its high sweetening power ascribed to its glycosides. Stevia also contains several bioactive compounds showing antioxidant, antiproliferative, antimicrobial, and anti-inflammatory activities. Since inflammation and oxidative stress play critical roles in the pathogenesis of many diseases, stevia emerges as a promising natural product that could support human health. In this study we set out to investigate the way stevia affects oxidative stress markers (e.g., SOD, CAT, GPx, GSH, MDA) in diseased rats administered stevia leaf extracts or glycosides. To this end, we performed an inclusive literature search, following PRISMA guidelines, and recruited multivariate meta-analysis and meta-regression to synthesize all available data on experimental animal models encountering (a) healthy, (b) diseased, and (c) stevia-treated diseased rats. From the 184 articles initially retrieved, 24 satisfied the eligibility criteria, containing 104 studies. Our results demonstrate that regardless of the assay employed, stevia leaf extracts restored all oxidative stress markers to a higher extent compared to pure glycosides. Meta-regression analysis revealed that results from SOD, CAT, GSH, and TAC assays are not statistically significantly different (*p* = 0.184) and can be combined in meta-analysis. Organic extracts from stevia leaves showed more robust antioxidant properties compared to aqueous or hydroalcoholic ones. The restoration of oxidative markers ranged from 65% to 85% and was exhibited in all tested tissues. Rats with diabetes mellitus were found to have the highest restorative response to stevia leaf extract administration. Our results suggest that stevia leaf extract can act protectively against various diseases through its antioxidant properties. However, which of each of the multitude of stevia compounds contribute to this effect, and to what extent, awaits further investigation.

## 1. Introduction

**Figure 1:**
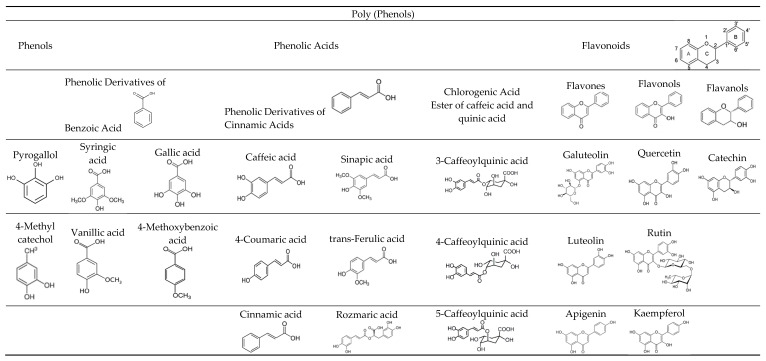
The main polyphenols found in *Stevia rebaudiana* (Bertoni) leaves.

*Stevia rebaudiana* Bertoni is a perennial shrub of the family Asteraceae which is endemic to northeastern Paraguay but also found in the nearby regions of Brazil and Argentina [1,2]. Stevia is mainly known for the high content of steviol glycosides in its leaves that are utilized as a non-sucrose and calorie-free sweetener in a variety of food products. Glycosides are organic compounds that consist of two parts, one part is a carbohydrate called glycone, which is connected via a glycoside bond to another part, the aglycone, a non-sugar group [3]. The best-known steviol glycosides are stevioside and rebaudioside A (RebA), which are the most abundant glycosides of the plant [4,5,6]. Additionally, other diterpene glycosides such as rebaudioside B, C, D, E, and F, steviolbioside, and dulcoside A also exist in leaves, but at significant lower concentrations. In addition to the sweet compounds, stevia leaves contain carbohydrates, lipids, dietary fibers, essential oils, water-soluble vitamins, minerals, and phenolic compounds [7,8]. Recent studies have shown several benefits of stevia leaf consumption on human health. Because of the high content of various phytoconstituents, stevia leaves appear to have a broad range of biological activities such as antidiabetic, antihypertensive, antimicrobial, anti-inflammatory, anti-tumor, and antioxidant activities [9,10]. Antioxidant activity in plants is most often due to a high content of polyphenols. The main polyphenols of stevia are phenols, phenolic acids, and flavonoids [11,12], as shown in (Figure 1). The main phenols are pyrogallol and 4-methylcatechol, while the main phenolic acids are derivatives of benzoic acid (syringic, vanillic, gallic, and 4-methoxybenzoic), cinnamic acids (caffeic, 4-coumaric, sinapic, trans-ferulic, and rosmaric), and chlorogenic acid (esters of caffeic and quinic acids) [13]. Flavonoids found in stevia leaves belong to three main groups, i.e., flavones (galuteolin, luteolin, apigenin), flavonols (quercetin, rutin, kaempferol), and flavanols (catechin) [14,15,16].

Oxidative stress in animals occurs when there is an imbalance between the production of reactive oxygen species (ROS) and the ability of the organism to neutralize or repair the resulting damage. ROS are highly reactive molecules that can damage cells and their components, such as DNA, proteins, and lipids. Some common ROS are the superoxide anion (O_2_^•^), hydrogen peroxide (H_2_O_2_), and the hydroxyl radical (^•^OH). Exposure of an animal body to high levels of ROS can lead to tissue damage and ultimately to the development of various degenerative diseases, such as cancer, cardiovascular disease, and neurodegenerative diseases [17]. Humans have several antioxidant defenses to counteract the harmful effects of ROS, including enzymes such as superoxide dismutase (SOD), catalase (CAT), and glutathione peroxidase (GPx), as well as other non-enzymatic antioxidant molecules such as glutathione (GSH) and vitamins C and E [18,19,20].

SOD catalyzes the conversion of the superoxide anion into hydrogen peroxide [21] (2O^2−^ + 2H^+^ → H_2_O_2_). Hydrogen peroxide is a substrate for CAT and GPx; CAT metabolizes hydrogen peroxide into harmless water and oxygen [22] (2H_2_O_2_ → 2H_2_O + O_2_), while GPx uses GSH to convert hydrogen peroxide and organic hydroperoxides into less harmful compounds, i.e., oxidized glutathione (GSSG) and water (2GSH + H_2_O_2_→ GSSG + 2H_2_O). GSH is a tripeptide molecule composed of three amino acids: glutamic acid, cysteine, and glycine. In its reduced form, GSH contains a thiol (-SH) group, and when GSH donates an electron to neutralize a free radical, it becomes oxidized and forms a disulfide bond (-S-S-) with another GSH molecule [23].

Lipid peroxidation is a process in which free radicals attack and damage lipids in cell membranes, leading to the production of reactive lipid peroxidation products such as malondialdehyde (MDA) and other harmful byproducts. MDA is considered a marker of lipid peroxidation and oxidative stress [24,25]. Lipid peroxidation can be triggered by a variety of factors, including oxidative stress, inflammation, and exposure to toxins or radiation. The process entails the attack of a free radical on a polyunsaturated fatty acid (PUFA) in the cell membrane [26], and the production of a peroxyl-radical lipid which is converted to MDA. MDA has been shown to have several toxic effects on the body, including DNA damage and alteration of proteins and enzymes, and it has been linked to a number of health problems, including inflammation, cancer, and cardiovascular disease [25].

Antioxidants are molecules that can contribute to the protection of cells from oxidative stress by neutralizing free radicals (ROS). Some common natural antioxidants are vitamins A, C, and E, as well as minerals such as selenium and zinc. Other natural antioxidants include phytochemicals, such as flavonoids and polyphenols, which are found in fruits, vegetables, and herbs [27]. Research results suggest that a diet high in antioxidants may protect against the harmful effects of oxidative stress [17,28,29]. The study of antioxidants and of plant extracts rich in antioxidants has emerged as an important and very potent research area on the role of oxidative stress in health and disease; it can provide qualitative and quantitative determination of their antioxidant capacity that can lead to the development of new natural-based treatments for modern-lifestyle diseases.

Studies in animal models have been used to investigate how diseases affect numerous markers of animal oxidation status. It has been reported that animal experimental models of various diseases are characterized by decreased levels of most enzymatic and non-enzymatic antioxidant markers, and by increased levels of MDA [19,24]; however, discrepancies among reported results do occur. The objective of the present study is to statistically combine all of the available data in the literature and determine the effect of stevia leaf extracts on oxidative stress markers in tissues of rats that have been infected with a disease and then (or in parallel) administered stevia extracts, mainly orally. The present meta-analysis is an effort to quantitatively synthesize all of the available data, uncover interchangeable methods, and summarize all of the existing evidence on the antioxidant impact of differently prepared stevia leaf extracts on several tissues of animals suffering from various diseases.

## 2. Materials and Methods

### 2.1. Literature Search Strategy and Eligibility Criteria

An all-inclusive literature search in the PubMed database (https://pubmed.ncbi.nlm.nih.gov/ (accessed on 26 January 2023) was carried out to retrieve all potential research articles exploring the antioxidant impacts of stevia, using the keywords ‘Stevia’, ‘antioxidant’, and ‘animal’ with their combinations and derivatives. The search was performed using the preferred reporting items for systematic reviews and meta-analyses (PRISMA) guidelines (http://www.prisma-statement.org/ (accessed on 26 January 2023)) along with the advice for best practices [30,31]. Screening of the reference lists of the included studies was also performed to incorporate all possible relevant publications. To eliminate publication bias and the implications of the grey literature, articles in various languages were taken into consideration [32]. Eligible criteria for inclusion in the meta-analysis were (a) intervention animal studies, (b) studies aimed at evaluating the effects of stevia leaves or stevia glycosides on various diseases, (c) use of control animals. Unrelated articles, in vitro studies, studies conducted on humans, observational studies, and reviews were excluded. We also excluded studies that did not provide sufficient information or data necessary for the analysis in order to ensure the reliability and validity of the results.

### 2.2. Data Extraction and Antioxidant Markers

Initially, titles and abstracts of the articles were screened, and relevant articles were further examined following the inclusion and exclusion criteria. The search results were assessed by two separate researchers (MP and PK), any discrepancies were discussed with GB and PB and decided upon by consensus. Upon reading all articles, it was found that the determination of oxidative stress was performed mainly with six assays measuring enzymatic activity, and levels of oxidative stress was expressed quantitatively in units of the six oxidation markers. The majority of the studies reported data for the following assays: (a) superoxide dismutase (SOD), (b) catalase (CAT), (c) glutathione peroxidase (GPx), (d) reduced glutathione (GSH), (e) malondialdehyde (MDA), and (f) total antioxidant capacity (TAC) [33].

All the above-mentioned methods by which oxidative stress can be estimated fall into three main categories [34]: (a) assays that measure the activity of antioxidant enzymes, (b) assays that measure lipid peroxidation, and (c) assays that measure total antioxidant capacity (TAC).

Assays used to measure antioxidant enzyme activity include SOD, CAT, and GPx. The activity of SOD [35] can be measured using a colorimetric assay that detects the amount of hydrogen peroxide produced by the action of SOD using formazan dye as an indicator. Formazan dye is measured colorimetrically at 560 nm. Superoxide anions react with formazan salts to produce a dye which can be detected colorimetrically. The greater the activity of SOD in the sample, the less formazan dye is produced. The activity of CAT [36,37] can be measured using a colorimetric assay that detects the rate of decomposition of hydrogen peroxide. The technique involves the reduction of dichromate in acetic acid to chromic acetate in the presence of hydrogen peroxide, which forms an impermanent intermediate perchromic acid. The amount of chromic acetate produced in the reaction is in direct proportion to the concentration of hydrogen peroxide employed. The chromic acetate produced is measured colorimetrically at 570 nm (Cr_2_ O_7_ ^−2^+ 7H_2_O_2_ → 2CrO_8_ ^−3^ + 5H_2_O + 4H^+^ followed by 2CrO_8_ ^−3^ + 6CH_3_COO^−^ + 6H ^+^ → 2Cr(CH_3_ COO)_3_ + 14H_2_O). The activity of GPx can be measured using a colorimetric assay that detects the amount of GSSG produced [38], which is coupled to the oxidation of NADPH to NADP^+^. The decrease in NADPH is proportional to the GPx activity and is monitored spectrophotometrically at 340 nm. When GSH reacts with 5,5′-dithiobis(2-nitrobenzoic acid) (DTNB) a yellow product is formed proportional to the GSH concentration that can be measured colorimetrically according to Ellman’s assay [39].

The extent of lipid peroxidation can be measured using a colorimetric assay that detects the amount of malondialdehyde (MDA) produced because of lipid peroxidation [25]. MDA reacts with thiobarbirutic acid (TBA), and the product is detected by the absorbance at 532 nm.

The total antioxidant capacity (TAC) assay [20] is an assay that measures the overall ability of a substance or biological sample to neutralize free radicals. It measures either the combination of both small molecule antioxidants and proteins, or the presence of small molecules alone that are present in the sample. This assay can be performed using various methods and, in the studies included herein, it was measured colorimetrically. The ferric reducing antioxidant power (FRAP) assay [40] is based on the ability of antioxidants to reduce ferric ion Fe^+3^ to ferrous ion Fe^+2^ in a redox reaction, the Fe^+2^ then reacts with the colorimetric reagent TPTZ (2,4,6-tripyridyl-s-triazine, and iron(III) chloride hexahydrate) to produce a complex which can be measured spectrophotometrically at 593 nm. In one study [41], TAC was indirectly measured by determining the residual H_2_O_2_ through the conversion of 3,5, dichloro dicloro-2-hydroxy benzensulphonate to a colored product. The measurement of this colored product can be measured spectrophotometrically at 500–510 nm.

Oxidative stress markers data were used to determine the effect of differently prepared stevia leaf extracts on different tissues of variously diseased animals.

In the studies included herein, dried leaves of stevia were ground to powder. The solvents used for the extraction included water or organic solvents (ethanol, acetone, or methanol). For hydroalcoholic extracts, solutions containing 70% to 80% methanol or ethanol were used. Generally, extractions took place with maceration and incubation to various temperatures [42,43] for different times (five min to 24 h) or using the Soxhlet extraction technique [44]. Subsequently, the extraction products were filtered and evaporated to complete dryness under reduced pressure using vacuum rotary evaporation. The resulting powder of the extracts was diluted with either distilled water or saline solutions for oral administration. The glycosides used in the studies reported herein were purchased ensuring that purities were >96%. In one study [45], the isolation and purification of the glycosides were performed using a diode array detector (JASCO HPLC system).

Data extraction was performed in a predetermined Microsoft Excel^®^ sheet. From each study, the following information was extracted: first author’s last name, publication year, country, type of assay determining oxidative stress, treatment, number of experimental animals, tissue of rats, type of disease, and the type of stevia leaf extracts or steviol glycosides. Antioxidant marker data were divided into three groups as follows: ‘control’, consisting of healthy normal rats; ‘case’, consisting of diseased rats; and ‘stevia’, the group with the diseased rats that received stevia extracts.

### 2.3. Statistical Analysis

The primary outcome of this meta-analysis was the standardized mean differences in oxidative stress marker estimates between the three animal groups, which is referred to as Cohen’s d. Secondary outcomes included the stratification analysis of oxidative stress markers’ performance in different tissues of rats, types of diseases, and types of stevia extracts. The model we used was based on the standard model of multivariate meta-analysis method [46,47,48]. We were interested in comparing the mean difference of the oxidative stress marker estimates between the three animal groups, measured in study *i* = 1,2,…k. The mean differences in oxidative stress marker estimates were used to determine the contrasts ‘control’ vs. ‘case’, ‘stevia’ vs. ‘case’, and ‘stevia’ vs. ‘control’, and were estimated using:

(1) $${\hat{d}}_{1i}=\frac{{\bar{X}}_{1i}-{\bar{X}}_{0i}}{S_{pooled,i}}\;\mathrm{and}\;{\hat{d}}_{2i}=\frac{{\bar{X}}_{2i}-{\bar{X}}_{0i}}{S_{pooled,i}}$$

where *X*_1*i*_, *X*_2*i*_, and *X*_0*i*_ are the means of the measured values of the oxidative stress markers in the ‘control’, ‘case’, and ‘stevia’ groups, respectively. *S_pooled,i_* is the pooled standard deviation in study *i*, given by:

(2) $$S_{pooled,i}=\sqrt{\frac{\left(n_{0i}-1\right)S_{0i}^{2}+\left(n_{1i}-1\right)S_{1i}^{2}+\left(n_{2i}-1\right)S_{2i}^{2}}{n_{0i}+n_{1i}+n_{2i}-3}}$$

with *n*_1*i*,_ *n*_2*i*_, and *n*_0*i*_ being the sample size of each group and *S*_1*i*_, *S*_2*i*_, and *S*_0*i*_ the standard deviation of the measured values for each group.

The variance of the effect size estimates *d*_1*i*_ and *d*_2*i*_ is estimated by:

(3) $$v\hat{a}r\left({\hat{d}}_{1i}\right)=s_{1i}^{2}=\frac{1}{n_{1i}}+\frac{1}{n_{0i}}+\frac{{\hat{d}}_{1i}{}^{2}}{2n_{i}},\;v\hat{a}r\left({\hat{d}}_{2i}\right)=s_{2i}^{2}=\frac{1}{n_{2i}}+\frac{1}{n_{0i}}+\frac{{\hat{d}}_{2i}{}^{2}}{2n_{i}}$$

and the covariance between the estimates of *d*_1*i*_ and *d*_2*i*_ is

(4) $$c\hat{o}v\left({\hat{d}}_{1i},{\hat{d}}_{2i}\right)=\frac{\;1}{n_{0i}}+\frac{{\hat{d}}_{1i}{\hat{d}}_{2i}\;}{2n_{i}}$$

with *n_i_* = *n_0i_* + *n_1i_*+ *n_2i_* being the total sample size of the study. The calculation of the within-studies covariance is very important since it plays a crucial role in the multivariate method.

Several statistical methods have been proposed to handle the issues of small sample sizes efficiently and to calculate accurate *p*-values and confidence intervals. The *d* can be corrected using the so-called Hedges’ **g**, which generates an unbiased estimate (the standardized mean difference d has the tendency to overestimate the absolute value in small samples), and the meta-analysis was conducted as proposed in [49]. In the multivariate random-effects meta-analysis, we assume that **g***_i_* = (**g**_1*i*_, **g**_2*i*_) is distributed following a multivariate normal distribution around the true means, according to the marginal model:

(5) $$g_{i}\sim MVN\left(g,C+\Sigma_{i}\right)$$

By Σ*_i_* we denote the within-studies covariance matrix:

(6) $$\Sigma_{i}=\left[\begin{array}{cc} s_{1i}^{2} & \rho_{W}s_{2i}s_{1i}\\ \rho_{W}s_{2i}s_{1i} & s_{2i}^{2} \end{array}\right]$$

The diagonal elements of Σ*_i_* are the study-specific estimates of the variance (Equation (3)), whereas the off-diagonal elements correspond to the pairwise within-studies covariances (Equation (4)), for instance, $\mathrm{cov}\left(g_{1i},g_{2i}\right)=\rho_{w}s_{1i}s_{2i}$. It is important to note that the elements of Σ*_i_* are considered known quantities. In contrast, by **C** we denote the between-studies covariance matrix, which is estimated during the fitting process:

(7) $$C=\left[\begin{array}{cc} \tau_{1}^{2} & \rho_{B}\tau_{1}\tau_{2}\\ \rho_{B}\tau_{1}\tau_{2} & \tau_{2}^{2} \end{array}\right]$$

The particular model takes into account the within-studies covariance and the between-studies covariance of the random terms, which is estimated in the model fitting procedure. A major advantage of the multivariate meta-analysis model is that it can accommodate studies reporting only one of the parameters of interest, resulting in borrowing strength from external studies. Another advantage is that with the estimated variance–covariance matrix, it can be used to perform global tests for the effect sizes where the estimates of both **g**_1_ and **g**_2_ influence all outcomes (‘control’ vs. ‘case’, ‘stevia’ vs. case’, and ‘stevia’ vs. ‘control’).

Moreover, to determine whether different oxidative stress markers can be combined in a meta-analysis, meta-regression analysis [50,51] was also used. This allowed us to assess whether studies that used different markers tended to have comparable data [46,52,53]. Multivariate meta-analysis and meta-regression analysis were performed using the statistical software package Stata13 [54]. In all tests, *p* ≤ 0.05 was used as the decision rule for significance testing. Meta-analysis was performed when two or more studies were available.

## 3. Results

### 3.1. Study Selection and Characteristics

**Figure 2:**
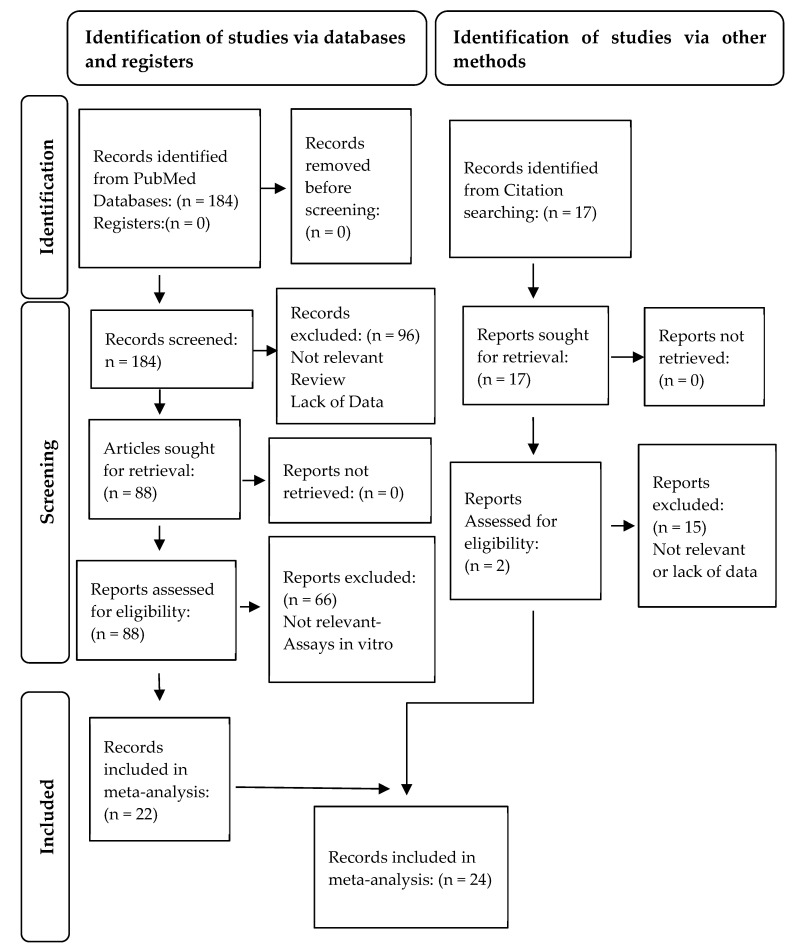
Flow diagram of systematic review to retrieve the selected studies for meta-analysis in accordance with the PRISMA statement.

A thorough literature search for antioxidant activity of stevia resulted in 184 articles. After screening of titles and abstracts, and compliance with the PRISMA guidelines (http://www.prisma-statement.org/ (accessed on 26 January 2023)) (Figure 2), 22 articles were found to satisfy the eligibility criteria [41,42,44,45,55,56,57,58,59,60,61,62,63,64,65,66,67,68,69,70,71,72]. Seventeen records were additionally retrieved from screening lists of references, and two of them [37,73] were enrolled in the meta-analysis, making a total of 24 articles, which contained 104 studies in total.

All the selected studies evaluated the effects of stevia extracts or stevia glycosides on rats as experimental models. The included studies predominantly reported data on SOD (21 studies), CAT (17 studies), and GPx (4 studies) activity, the content of GSH (27 studies), the assessment of TAC (2 studies), and the content of MDA (33 studies), all of which are shown in Table 1. Data on oxidative stress markers concerned 13 rat tissues, including liver, kidney, pancreas, heart, serum, plasma, skeletal muscles, brain, ovary, colon, duodenum, jejunum, and ileum. Antioxidant activity was investigated in 13 types of diseases, comprising diabetes mellitus, liver diseases, renal disorders, ulcerative colitis, metabolic syndrome, polycystic ovary syndrome, and epilepsy. Four types of stevia extracts were tested for their effect after administration: aqueous, organic, hydroalcoholic, and fractions of methanolic extracts. Hence, overall, our data enrolled 15 studies with aqueous extracts, 19 studies with organic extracts, 40 studies with hydroalcoholic extracts and only 1 with methanolic fractions of extract. In addition, 20 studies were performed with stevioside, 5 studies with rebaudioside A, and 1 study with extracted sweeteners from stevia leaves. All the selected studies investigated the effects of oral administration of stevia extracts or steviol glycosides in rats as a treatment, except for two articles in which administration was performed intraperitoneally. In three of the articles [61,71,72], the use of stevia was investigated as a pre-treatment prior to the induction of a disease, while in all other studies, stevia was administered after the disease had been induced.

The administration of the substance varied in terms of the doses employed, with doses ranging from 2 mg/kg to 300 mg/kg for pure glycosides and from 80 mg/kg to 500 mg/kg for leaf extracts. Additionally, the period of treatment varied from one to twelve weeks. It should be noted that the studies were conducted in six countries: Mexico, Egypt, Saudi Arabia, Iran, China, and India. The characteristics of the selected studies that were encountered in the meta-analysis are presented in Table 1.

### 3.2. Bioactive Compounds from Stevia Leaf Extracts Exert Significantly Higher Antioxidant Activity Compared to Stevia Glycosides

To assess the effect of stevia whole leaf extracts or steviol glycosides on diseased animals, the differences of oxidative stress marker measurements between control and cases were enrolled in a multivariate meta-analysis for the assays SOD, CAT, GPx, GSH, TAC, and MDA. If stevia could reverse the effect of the ‘disease’ in case animals, in terms of oxidative stress markers, then the differences ‘ ‘control–case’ and ‘stevia case’ would be almost the same; consequently, the difference ‘stevia – control’ in assays’ values would be very low and perhaps non-significant.

Overall, the meta-analysis results for each separate assay (SOD, CAT, GPx, GSH, CAT, MDA) showed that differences for the contrasts ‘control–case’ were a bit higher than the differences ‘stevia–case’ (for the MDA assay absolute values were considered). The differences ‘stevia–control’ were significantly lower, and importantly, in the GPx assay, this difference was not even statistically significant (difference −6.03, *p*-value 0.22) (Table 2), supporting our previous hypothesis. The above data suggest that stevia administration can, at least partially, restore the oxidation markers of diseased rat tissues.

**Figure 3:**
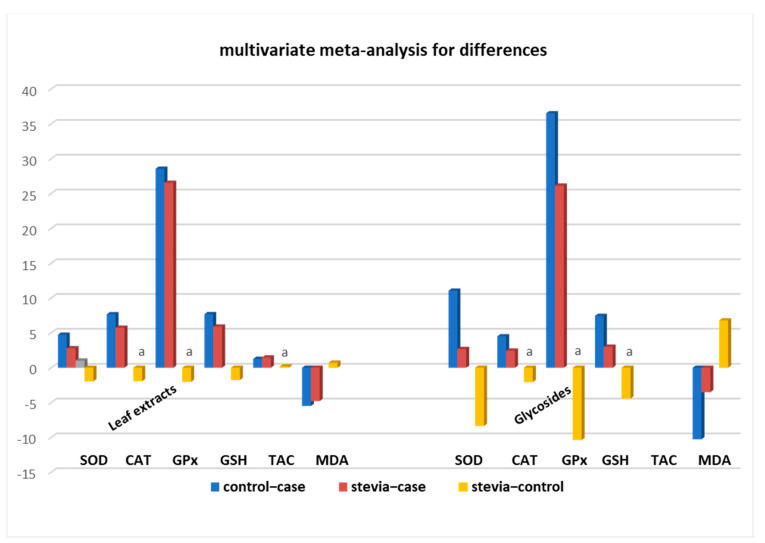
Stevia whole leaf extracts can better restore antioxidant markers of diseased animal tissues compared to pure steviol glycosides. a: non-statistically significant difference between compared groups.

Stratification analysis of all of the assays’ measurements according to the administered leaf extracts or compounds revealed that with stevia glycosides the differences ‘control–case’ were much higher (in absolute values) than the differences ‘stevia–case’ (SOD: 11.04 vs. 2.67, GSH: 7.42 vs. 2.99, and MDA: −10.28 vs. −3.51) suggesting that stevia glycosides cannot restore antioxidant activity to the extent that whole leaf extracts can (Table 2 and Figure 3). This is further verified by the fact that ‘stevia–control’ differences (for SOD, GSH, and MDA assays) were much higher (absolute values) than the ‘stevia–case’ differences in the ‘glycosides’ datasets (Table 2). In the CAT and GPx assays, all of the differences were not statistically significant, perhaps due to the limited number of studies meaning that they could not provide a robust result. Importantly, significant restoration of antioxidant activity of diseased animals was observed with administration of whole leaf extracts in studies performed with CAT and GPx assays, as suggested by the non-statistically significant differences in the ‘stevia–control’ values (*p*-values 0.08 and 0.70), respectively. Moreover, the fact that significant restoration of antioxidant activity by stevia leaf extracts was seen with the SOD, GSH, TAC, and MDA assays to 59%, 77%, 111%, and 87%, respectively, further supports our findings that whole stevia leaf extracts can better restore oxidation markers in experimental animals compared with pure stevia glycosides, which showed restoration ability ranging from 24% (SOD) to 71% (GPx) (Table 2 and Figure 3).

Moreover, meta-regression analysis for the datasets from the SOD, GSH, TAC, and MDA assays testing leaf extracts and glycosides was also employed and revealed a statistically significant dependence of the results on the type of treatment (leaf extract or glycosides) with a *p*-value = 0.0023. Taken together, the results above indicate that, in all assays, treatment with whole leaf extract of stevia exhibited a remarkable restorative potential as opposed to treatment with isolated glycosides. Consequently, further analyses were performed with whole leaf extracts.

### 3.3. Datasets from SOD, CAT, GSH, and TAC Assays Can Be Combined for Meta-Analysis: Meta-Regression Analysis

We next wondered whether we could synthesize the results of all experiments, despite the different values each exhibited, in order to perform multivariate meta-analysis on the combined data from all assays. Towards this, multivariate meta-regression analysis for leaf extract datasets on the differences ‘control–case’ and ‘stevia–case’ from all assays was executed and revealed a relationship between the assay method and their outcomes (*p* = 0.000), with GPx and MDA results being the assays with statistically significant differences compared to the rest of them (coefficient = 6.14, *p* = 0.005; and coefficient = −10.95, *p* = 0.000, respectively, and constant = 5.31 for the difference ‘control–case’). Subsequently, meta-regression analysis of leaf extract datasets on the differences ‘control–case’ and ‘stevia–case’ for the rest of the assays, i.e., SOD, CAT, GSH, and TAC revealed the absence of any relationship between assay results and the type of assay (*p* = 0.184). As a result, datasets on the differences ‘control–case’ and ‘stevia–case’ from the above four assays can be treated as coming from the same source and can be combined in consequent meta-analyses.

### 3.4. Stratification Meta-Analysis for Datasets from Leaf Extracts

**Figure 4:**
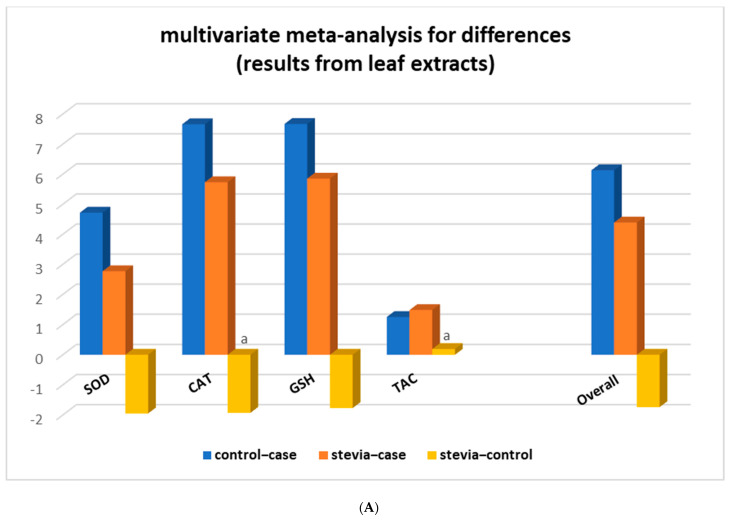

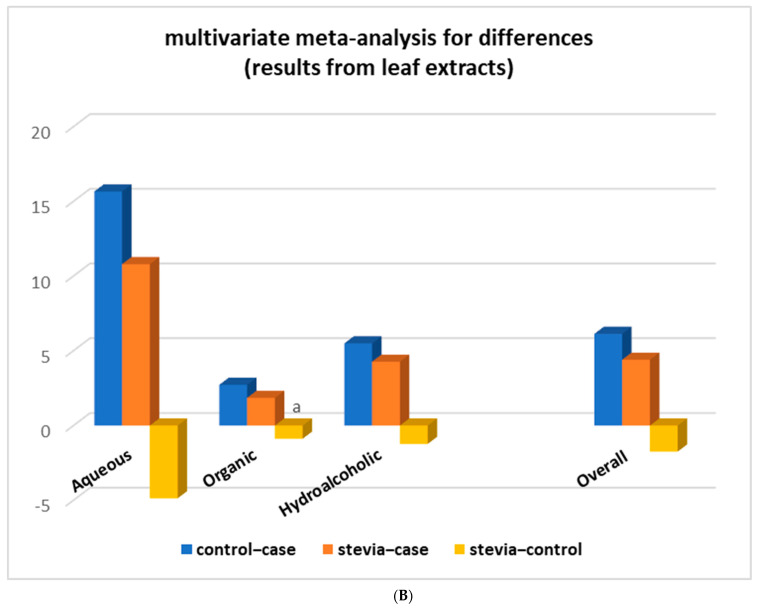
Stratification of the meta-analysis according to (**A**) type of assay and (**B**) type of leaf extract. a: non-statistically significant difference between compared groups. Overall: results from combined SOD, CAT, GSH, and TAC assays.

Multivariate meta-analysis was then performed with the combined leaf extract datasets from four assays (SOD, CAT, GSH, and TAC) comprising 50 studies in which rats had only leaf extracts administered. Analysis showed that in all assays, the ‘stevia–control’ differences were lower than ‘control–case’ and ‘stevia–case’, and in the CAT and TAC assays, they were not even statistically significant (Figure 4A and Table 3).

Stratification analysis according to the type of stevia extract revealed that organic extracts were the most potent in restoring the antioxidant activity in diseased rats administered with various types of leaf extracts (Table 3). The difference ‘stevia – control’ was not statistically significant (d = −0.88 with *p*-value = 0.13) for the combined all four assays datasets, nor for each assay separately. The same ‘stevia–control’ differences for aqueous and hydroalcoholic types of extracts, though statistically significant, were still much lower than the other differences (Figure 4B and Table 3). In addition, stratification by type of extract within the CAT assay results showed that treatment with aqueous stevia leaf extracts can also completely restore the CAT oxidative stress marker of diseased animals to the levels of the control animals as the differences ‘stevia – control’ were not statistically significant, i.e., for the aqueous (d = −6.76 and *p*-value = 0.36), for the organic (d = −1.09 and *p*-value = 0.29), and for the hydroalcoholic extracts (d = −1.09 and *p*-value = 0.49) (Table 3). The four studies that investigated the effect of stevia leaf extracts on diseased rats with the GSH assay showed that only organic stevia leaf extracts restored GSH values of diseased animals (d = 0.14 and *p*-value = 0.83). Similarly, no significant difference was observed for the ‘stevia–control’ contrast in the SOD assay when organic extracts were administered to diseased rats.

**Figure 5:**
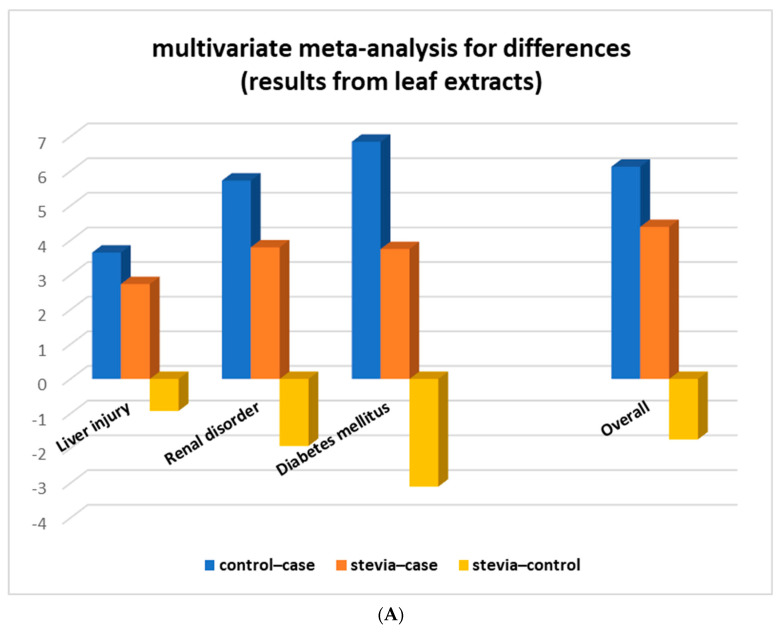

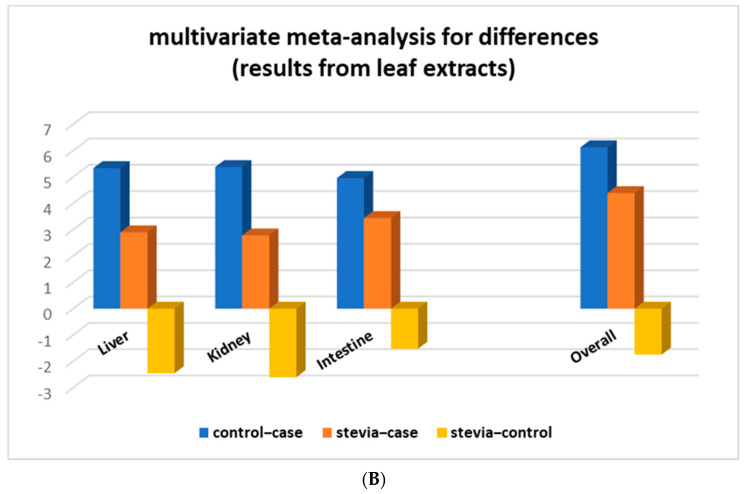
Stratification of the meta-analysis according to (**A**) type of disease and (**B**) type of tissue. Overall: results from combined SOD, CAT, GSH, and TAC assays.

We next stratified our analysis according to the different types of disease that rats had suffered from (Table 4). The data were grouped into three main categories: liver injury (acute liver injury, hepatotoxic, liver cirrhosis, liver fibrosis), renal disorder (hyperuricemia, renal ischemia/reperfusion), and diabetes mellitus. The results of the multivariate meta-analysis demonstrated full restoration of antioxidant SOD, CAT, and GSH activity of diseased rats suffering from diabetes mellitus as the differences ‘stevia – control’ were not statistically significant (SOD: d = −0.14 with *p*-value = 0.94, CAT: d = −0.14 with *p*-value = 0.94, and GSH: d = −1.73 with *p*-value = 0.06). For diseased rats that suffered from liver injury or renal disorder, the multivariate meta-analysis results revealed a significant restoration, though not full, of the oxidative stress markers when they were administered stevia leaf extracts (Table 4 and Figure 5A).

We next wondered whether specific rat tissues or cell types could have been affected differently from stevia leaf extract administration to the experimental animal. Thus, we stratified our analysis on the combined (only leaf extract) datasets from the four assays (SOD, CAT, GSH, and TAC) according to different types of animal tissue (Table 5). The results revealed that complete restoration of the antioxidant enzyme SOD’s activity was seen in liver samples, since d = −0.32 with *p*-value = 0.67. However, it should be noted that in all types of tissues, the values of the differences ‘stevia – control’ suggested a significant restoration of the oxidative stress markers of the diseased animals when receiving stevia leaf extracts (Table 5 and Figure 5B). Taken together, the above results support that stevia leaf extracts do exert antioxidant activity on diseased animals and especially to those that suffer from diabetes mellitus.

### 3.5. Meta-Analysis of Lipid Peroxidation (MDA) Assay Datasets

MDA is a lipid peroxidation product, and thus, the MDA assay is used as a marker for oxidative stress, the more the oxidative stress, the higher the MDA assay values. Thus, control experimental animal tissues show low MDA levels while diseased animals present higher MDA levels. Due to this particularity and because of the different biochemical principle that this assay is based on, MDA estimates could not be directly compared to the previously analyzed oxidation markers, and MDA results could not be analyzed together with the rest.

It is important to note here that whole leaf extracts reverted the oxidation status of diseased experimental animals while pure glycosides did not, as shown in Table 2 (difference ‘stevia–control’ leaf extract: 0.71, *p*-value 0.00; glycosides: 6.77, *p*-value 0.01). Consequently, a meta-analysis for stevia leaf extract datasets from 25 studies performed with the MDA assay showed significant restoration of the oxidation status of diseased rats when administered stevia leaf extracts of 87.07% (from −5.49 (*p* = 0.00) to −4.78 (*p* = 0.00)) (Table 6). It is worth noting that although the ‘stevia–control’ difference is statistically significant, the values of the differences between the ‘control’ and ‘case’ groups and the ‘control’ and ‘stevia’ groups decreased significantly.

Next, we stratified the multivariate meta-analysis of the MDA assay datasets according to type of stevia leaf extract, tissue, and disease and revealed that the restoration of the MDA content of samples of more than 70% was seen under every tested parameter. In particular, diseased rats administered organic extracts showed that the difference ‘stevia–control’ was not statistically significant (d = 0.62, *p*-value = 0.48), connotating a complete oxidative status restoration. This finding is in accordance with results from other assays (SOD, CAT, GSH, TAC) verifying that organic extracts possess high antioxidant potency. 

Stratification of the meta-analysis according to the disease type (Table 6) highlighted that in diabetes, the low MDA levels of diseased animals are completely restored after leaf extract administration (‘stevia–control’ −0.41, *p* = 0.12). This observation supports our previous finding, that with all tested assays (SOD, CAT, GSH) whole stevia leaf extract administration completely restores all antioxidant markers levels, especially in diabetic experimental animals (Table 4).

Importantly, the ‘stevia–control’ differences were all found to not be statistically significant for all tissues tested, apart from liver (kidney: d = 0.68 (*p*-value = 0.13), duodenum–jejunum–ileum: d = 0.35 (*p*-value = 0.23), pancreas: d = 0.02 (*p*-value = 0.95)). Measurements in liver tissue showed a marginally statistically significant difference between the control and stevia groups (*p* = 0.04); however, restoration of the MDA measurements was up to 85% for the leaf-extract-treated diseased animals.

Taken together the above data show that even if complete restoration of antioxidant markers was not statistically significantly accomplished in diseased rats that were administered stevia, partial restoration did occur. As shown in Figure 3 this restoration rises from 65% to 85% for all antioxidant markers, reinforcing the notion that stevia leaf extracts do possess significant antioxidant activity when administered orally.

## 4. Discussion

Recent research towards sustainable solutions for global health is guided from the need to produce natural products with efficient health promoting properties that present a low incidence of side effects. Stevia is an aromatic plant known as a no-calories sweetener. By the identification of many bioactive compounds contained in its leaf extracts, stevia has emerged as a health beneficial aromatic herbal, characterizing it as high-added-value plant within the agrifood sector [69,74,75]. The antioxidant potency of stevia is currently being extensively studied with a variety of approaches (direct or indirect) entailing various (bio)chemical principles and cascades, using in vitro and in vivo systems. The present meta-analysis is the first effort to quantitatively synthesize all of the published evidence and assess the antioxidant effect of stevia phytoconstituents when administered to experimentally diseased animals.

Our findings indicate a statistically significant antioxidant restorative effect of oxidative status markers of experimentally diseased animals after they have been administered stevia leaf extracts. By combining data from 24 articles comprising 104 individual studies, we show this restorative activity is mainly exerted by stevia whole leaf extracts and to a minor extent by pure glycosides. Our findings are based on datasets coming from six different methods (SOD, CAT, GPx, GSH, TAC, and MDA) measuring either antioxidant enzyme activities (SOD, CAT, GPx) or products linked to the activities of antioxidant enzymes, or even oxidation products such as MDA, which measures the lipid peroxidation status of a cell. A stratification analysis of the stevia whole leaf extract datasets revealed a robust antioxidant effect of organic extracts of stevia leaves. Moreover, diabetes mellitus emerged as the disease with the highest restorative response to stevia leaf extract administration. Concerning the tissues on which the oxidative markers were tested, all showed significant restoration, from 65% to 85%, regardless of the type of assay employed.

The challenge that we faced in the present study was not only to summarize the evidence and draw conclusions for a positive relationship between stevia consumption and antioxidant activity, but to synthesize measurements coming from three different situations from six different assays on tissues from experimental rats. 

Firstly, since we had to compare values from more than two animal groups (control, diseased, and stevia-treated) we employed multivariate meta-analysis [46,48,49]. This approach enabled us to perform multiple comparisons avoiding the need for the usual adjustments in order to avoid type I errors. Moreover, employing multivariate meta-analysis allowed us to obtain the estimated covariant matrix from which we could estimate the confidence intervals of the differences we measured and utilize the statistical significance of the differences [49,76].

The second challenge we faced was whether we could combine datasets from some or all assays in order to obtain results with more power. For this, multivariate meta-regression analysis was recruited to control for potential effect modifiers or any association between group differences and various assays [77,78]. The assay results were significantly dependent on the MDA assay (as expected), and on the GPx method as well. Interestingly, no dependence on the method was seen when meta-regression was employed with datasets from the SOD, CAT, GSH, and TAC assays. Given that the SOD, CAT, GPX, and GSH assays measure antioxidant enzyme activities and all act in the same pathway, i.e., one with being downstream from another, one would expect that their datasets could be combined. Employing multivariate meta-regression, we proved this assumption as correct; however, given the available data, only the SOD, CAT, and GSH assay datasets could be combined. In addition, we proved that the TAC assay datasets could also be pooled to obtain more robust estimates. Collectively, multivariate meta-regression meta-analysis enabled us to pool data from different sources, thus increasing the power of our analysis, a result that would not be possible to obtain under a unified/univariate meta-analysis [49].

Our initial hypothesis, that both stevia leaf extracts and pure stevia glycosides could possess antioxidant activity, though to different extents, appears to have a biological basis. Polyphenolic compounds from a plethora of plant resources have repeatedly been shown to display antioxidant potential [79,80,81] in direct assays [20,82] and exert effective antioxidant activity *in vivo*, including cell lines [20], experimental animals [83], or even humans [84]. Our findings suggest that stevia glycosides do possess a limited antioxidant activity. Glycosides are complex molecules, constituting a sugar molecule (glycone) bound to another functional group (aglycone), found in many living organisms. Although sugars have never been reported to convey any antioxidant activity, other functional groups may possess various functions. The aglycone in stevia glycosides is the diterpene steviol that is the major metabolite produced in the lower gastrointestinal tract of mammals [3]. Thus, the limited antioxidant activity seen with stevia glycosides could be attributed to the diterpene steviol. This is in accordance with previous findings showing that a combination of stevia glycosides could increase the SOD and CAT activities of rat cardiac fibroblasts when exposed to hydrogen peroxide [85]. The antioxidant properties of stevia glycosides were also observed with direct assays on food and fruit beverages [86,87]. Moreover, steviol glycosides were found to restore oxidative stress status in fish [88].

We acknowledge that the present meta-analysis possibly has some limitations that could have affected the integrity of our results. The studies recruited in the present meta-analysis covered a diverse range of stevia extracts or pure glycoside dosages and time settings. The variability in the interventions concerning the time that the intervention started (before or after the animals were diseased) and the time that intervention started after the induction of disease, together with limited information given in each study, made it impossible to stratify according to dosage. A meta-regression analysis to estimate the association of antioxidant markers with extract preparation, dosage, or time of intervention would be of high value; yet, such data were not available from all studies to perform the corresponding tests. However, our analysis could account for the type of extracts given as intervention, the type of tissue, and the disease by stratifying our analysis accordingly. We could not account for intrinsic differences within studies that constitute confounding factors that can potentially influence the quality of the parameters assessed. For example, sample handling (time and temperature of sample storage before being assayed, homogenization protocols) or the different genetic backgrounds of rats used could have affected the assays’ outcomes, and consequently, partially, the results of the present meta-analysis. Nonetheless, we performed the meta-analysis carefully, taking all these considerations into account. Finally, despite our systematic approach and effort to include all existing studies, gray literature bias cannot be completely ruled out.

Increased stevia consumption has emerged from global awareness of (a) the risks of sugar-related diseases (obesity, diabetes); (b) the fact that synthetic sweeteners may lead to long-term health problems; and (c) that stevia, being a natural product, has, most probably, no long-term side effects. The bottleneck in the industrial use of stevia is the cost related to steviol glycoside extraction and purification. Sustainability issues have also emerged concerning stevia’s waste disposal [89]. The present meta-analysis is the first effort to quantitatively estimate the effect of stevia leaf extract consumption, in addition to steviol glycosides, highlighting an additional—yet unexploited—health promoting property that can give stevia (and perhaps stevia waste) a second chance to rescue human health and global sustainability. Only if uniform settings are used can we obtain accurate comparisons of types of stevia extracts, dosage, and time interventions, and more in vivo experiments can be performed in order to obtain accurate insights into, to what extent can human health benefit from stevia consumption.

## Figures and Tables

**Table 1:** Characteristics of the 104 studies included in the meta-analysis.

Author	Year	Country	Assay	After Treatment (A) Pre-Treatment (P) with Stevia	# Controls	Control Value	Control SD	# Cases	Case Value	Case SD	# Stevia	Stevia Value	Stevia SD	Extract/Compound	Type of Tissue Tested	Type of Disease
Mostafa et al. [72]	2020	Egypt	SOD	80 mg/kg/day orally, 1 week (A)	10	3.6	0.11	10	1.6	0.1	10	2.66	0.09	Aqueous	Colon	Ulcerative colitis
Mehmood et al. [64]	2019	China	SOD	400 mg/kg/day orally, 8 weeks (A)	8	118	6.1	8	53	2.8	8	107	12	Hydroalcoholic	Duodenum	Hyperuricemia
Mehmood et al. [64]	2019	China	SOD	400 mg/kg/day orally, 8 weeks (A)	8	70	7.9	8	30	7	8	51	12	Hydroalcoholic	Jejunum	Hyperuricemia
Mehmood et al. [64]	2019	China	SOD	400 mg/kg/day orally, 8 weeks (A)	8	55	8.1	8	21	4.5	8	40	4.5	Hydroalcoholic	Ileum	Hyperuricemia
Mehmood et al. [63]	2020	China	SOD	200 mg/kg/day orally, 4 weeks (A)	8	118	4.9	8	91	5.9	8	115	5	Hydroalcoholic	Serum	Hyperuricemia
El-Mesallamy et al. [45]	2018	Egypt	SOD	200 mg/kg/day orally, 4 weeks (A)	10	48	4	10	25	7.5	10	41	4	Hydroalcoholic	Skeletal muscles	Diabetes mellitus
El-Mesallamy et al. [45]	2018	Egypt	SOD	2 mg/kg/day orally, 4 weeks (A)	10	48	4	10	25	7.5	10	30	4	Stevioside	Skeletal muscles	Diabetes mellitus
Latha et al. [70]	2017	India	SOD	500 mg/kg/day orally, 1 week (A)	8	21	4.24	8	4	4.52	8	16.5	2.54	Hydroalcoholic	Liver	Acute liver injury
Latha et al. [70]	2017	India	SOD	250 mg/kg/day orally, 1 week (A)	8	21	4.24	8	4	4.52	8	17	4.52	Stevioside	Liver	Acute liver injury
Moselhy et al. [66]	2016	Saudi Arabia	SOD	200 mg/kg/day orally, 2 weeks (A)	10	0.25	0.0023	10	0.18	0.03	10	0.21	0.029	Organic	Liver	Hepatotoxic
Perumal et al. [67]	2016	India	SOD	100 mg/kg/day orally, 3 weeks (A)	6	10.15	1.05	6	4.63	0.95	6	6.75	1.25	Hydroalcoholic	Liver	Diabetes mellitus
Perumal et al. [67]	2016	India	SOD	100 mg/kg/day orally, 3 weeks (A)	6	11.04	1.3	6	4.63	1.69	6	6.74	1.18	Hydroalcoholic	Kidney	Diabetes mellitus
Shivanna et al. [71]	2012	India	SOD	NR orally, 4 weeks (P)	10	2.41	0.91	10	1.21	0.05	10	2.72	0.52	Fraction methanol	Liver	Diabetes mellitus
Myint et al. [73]	2020	China	SOD	12 mg/kg/day orally, 6 weeks (A)	6	74.53	1.82	6	36.61	1.44	6	39.23	1.28	Rebaudioside A	Liver	Diabetes mellitus
Myint et al. [73]	2020	China	SOD	10 mg/kg/day orally, 6 weeks (A)	6	74.53	1.82	6	36.61	1.44	6	44.46	1.28	Stevioside	Liver	Diabetes mellitus
Singh et al. [37]	2013	India	SOD	300 mg/kg/day orally, 3 weeks (A)	7	50.5	32.80	7	37.5	18.79	7	50	10.85	Organic	Liver	Diabetes mellitus
Singh et al. [37]	2013	India	SOD	300 mg/kg/day orally, 3 weeks (A)	7	190.2	50.27	7	40	19.84	7	10	14.02	Organic	Pancreas	Diabetes mellitus
Singh et al. [37]	2013	India	SOD	300 mg/kg/day orally, 3 weeks (A)	7	55	9.26	7	25	9.26	7	9	9.26	Organic	Kidney	Diabetes mellitus
El-Hadary et al. [60]	2021	Egypt	SOD	300 mg/kg/day orally, 8 weeks (A)	10	54.3	2.3	10	47.2	1.2	10	58.8	0.9	Hydroalcoholic	Liver	Diabetes mellitus
Morsi et al. [65]	2022	Egypt	SOD	300 mg/kg/day orally, 4 weeks (A)	7	36.25	1.25	7	21.25	3	7	29	1.75	Glycosides-sweetener	Ovary	Polycystic ovary syndrome
Deenadayalan et al. [59]	2021	India	SOD	20 mg/kg/day orally, 45 days (A)	6	32.5	3.06	6	13	1.84	6	20	1.84	Stevioside	Skeletal muscles	Diabetes mellitus
Mostafa et al. [72]	2020	Egypt	CAT	80 mg/kg/day orally, 1 week (P)	10	7.95	0.11	10	3.9	0.08	10	6.6	0.09	Aqueous	Colon	Ulcerative colitis
Elsaid et al. [61]	2019	Egypt	CAT	200 mg/kg/day orally, 5 weeks (P)	12	34.66	3.14	12	14	2.36	12	27.66	3.72	Hydroalcoholic	Kidney	Renal ischemia/reperfusion
Abdallah et al. [55]	2022	Egypt	CAT	500 mg/kg/day orally, 1 week (A)	7	36	0.5	7	21	5	7	30	0.5	Organic	Liver	Liver disease
Abdallah et al. [55]	2022	Egypt	CAT	250 mg/kg/day orally, 1 week (A)	7	36	0.5	7	21	5	7	35	5	Stevioside	Liver	Liver disease
Mehmood et al. [63]	2020	China	CAT	200 mg/kg/day orally, 4 weeks (A)	8	17.5	1.5	8	8	1.1	8	10.5	1.2	Hydroalcoholic	Serum	Hyperuricemia
Moselhy et al. [66]	2016	Saudi Arabia	CAT	200 mg/kg/day orally, 2 weeks (A)	10	0.89	0.07	10	0.32	0.05	10	0.76	0.05	Organic	Liver	Hepatotoxic
El Nashar et al. [41]	2022	Egypt	CAT	200 mg/kg/day orally, 4 weeks (P and A)	10	67.67	31.53	10	52.08	29.6	10	95.25	35.45	Organic	Brain	Epilepsy
Hussein et al. [62]	2020	Egypt	CAT	400 mg/kg/day orally, 4 weeks (A)	8	17	0.45	8	7	0.19	8	22.5	0.98	Hydroalcoholic	Heart	Diabetes mellitus
Shivanna et al. [71]	2012	India	CAT	NR orally, 4 weeks (P)	10	1.02	0.05	10	0.52	0.07	10	0.78	0.17	Fraction methanol	Liver	Diabetes mellitus
Assaei et al. [44]	2016	Iran	CAT	400 mg/kg/day orally, 4 weeks (A)	10	29.4	8.85	10	9.9	6.96	10	33.7	6.33	Aqueous	Pancreas	Diabetes mellitus
Deenadayalan et al. [59]	2021	India	CAT	20 mg/kg/day orally, 45 days (A)	6	11.5	3.06	6	6.25	3.18	6	8.75	1.83	Stevioside	Skeletal muscles	Diabetes mellitus
Perumal et al. [67]	2016	India	CAT	100 mg/kg/day orally, 3 weeks (A)	6	42.8	6.2	6	25.8	3.72	6	35.69	6.42	Hydroalcoholic	Liver	Diabetes mellitus
Perumal et al. [67]	2016	India	CAT	100 mg/kg/day orally, 3 weeks (A)	6	34.04	5.27	6	23.56	1.54	6	25.3	1.71	Hydroalcoholic	Kidney	Diabetes mellitus
El-Mesallamy et al. [45]	2018	Egypt	CAT	200 mg/kg/day orally, 4 weeks (A)	10	90	1	10	55	3	10	80	4	Hydroalcoholic	Skeletal muscles	Diabetes mellitus
El-Mesallamy et al. [45]	2018	Egypt	CAT	2 mg/kg/day orally, 4 weeks (A)	10	90	1	10	55	3	10	72	4	Stevioside	Skeletal muscles	Diabetes mellitus
Latha et al. [70]	2017	India	CAT	500 mg/kg/day orally, 1 week (A)	8	0.87	0.73	8	0.21	0.03	8	0.51	0.31	Hydroalcoholic	Liver	Acute liver injury
Latha et al. [70]	2017	India	CAT	250 mg/kg/day orally, 1 week (A)	8	0.87	0.73	8	0.21	0.03	8	0.3	0.28	Stevioside	Liver	Acute liver injury
El-Mesallamy et al. [45]	2018	Egypt	GPx	200 mg/kg/day orally, 4 weeks (A)	10	515	2	10	280	4	10	480	7	Hydroalcoholic	Skeletal muscles	Diabetes mellitus
El-Mesallamy et al. [45]	2018	Egypt	GPx	2 mg/kg/day Orally, 4 weeks (A)	10	515	2	10	280	4	10	450	3.5	Stevioside	Skeletal muscles	Diabetes mellitus
Deenadayalan et al. [59]	2021	India	GPx	20 mg/kg/day orally, 45 days (A)	6	26	6.12	6	14	3.68	6	20	3.68	Stevioside	Skeletal muscles	Diabetes mellitus
El-Hadary et al. [60]	2021	Egypt	GPx	300 mg/kg/day orally, 8 weeks (A)	10	165.6	0.7	10	137.8	1.4	10	175.8	5.3	Hydroalcoholic	Liver	Diabetes mellitus
Mostafa et al. [72]	2020	Egypt	GSH	80 mg/kg/day orally, 1 week (A)	10	5.7	0.09	10	2.2	0.05	10	4.9	0.03	Aqueous	Colon	Ulcerative colitis
Abdel-Aal et al. [56]	2021	Egypt	GSH	400 mg/kg/day orally, 3 weeks (A)	8	22	3.11	8	3	0.70	8	12.5	0.70	Aqueous	Liver	Diabetes mellitus
Abdel-Aal et al. [56]	2021	Egypt	GSH	400 mg/kg/day orally, 3 weeks (A)	8	22	1.41	8	7	0.71	8	17	1.27	Aqueous	Kidney	Diabetes mellitus
Hussein et al. [62]	2020	Egypt	GSH	400 mg/kg/day orally, 4 weeks (A)	8	11.25	0.4	8	3.1	0.23	8	11.7	0.97	Hydroalcoholic	Heart	Diabetes mellitus
Mehmood et al. [64]	2019	China	GSH	400 mg/kg/day orally, 8 weeks (A)	8	125	37.5	8	40	7.5	8	100	27	Hydroalcoholic	Duodenum	Hyperuricemia
Mehmood et al. [64]	2019	China	GSH	400 mg/kg/day orally, 8 weeks (A)	8	100	27	8	40	2	8	80	17.5	Hydroalcoholic	Jejunum	Hyperuricemia
Mehmood et al. [64]	2019	China	GSH	400 mg/kg/day orally, 8 weeks (A)	8	100	8	8	35	10	8	84	10	Hydroalcoholic	Ileum	Hyperuricemia
Elsaid et al. [61]	2019	Egypt	GSH	200 mg/kg/day orally, 5 weeks (P)	12	8.65	0.57	12	3.38	0.36	12	7.17	0.64	Hydroalcoholic	Kidney	Renal ischemia/reperfusion
Ramos-Tovar et al. [43]	2019	Mexico	GSH	100 mg/kg/day orally, 12 weeks (A)	8	10	1.98	8	3.75	1.95	8	8.55	1.27	Aqueous	Liver	Liver cirrhosis
Casas-Grajales et al. [58]	2019	Mexico	GSH	20 mg/kg/twice daily intraperitoneally, 8 weeks (A)	8	13	0.85	8	10	0.28	8	13.5	1.13	Stevioside	Liver	Liver fibrosis
Casas-Grajales et al. [57]	2019	Mexico	GSH	20 mg/kg/twice daily intraperitoneally, 8 weeks (A)	8	13.3	0.85	8	10	0.28	8	12.2	0.57	Rebaudioside A	Liver	Liver fibrosis
Ramos-Tovar et al. [42]	2018	Mexico	GSH	100 mg/kg/day orally, 10 weeks (A)	8	5.6	1.41	8	3	0.28	8	4.5	1.56	Aqueous	Liver	Liver cirrhosis
Ramos-Tovar et al. [68]	2018	Mexico	GSH	100 mg/kg/day orally, 1 week (A)	8	11.5	1.27	8	6	2.55	8	11	2.55	Aqueous	Liver	Liver cirrhosis
Latha et al. [70]	2017	India	GSH	500 mg/kg/day orally, 1 week (A)	8	230	14.14	8	95	28.29	8	212	98.99	Hydroalcoholic	Liver	Acute Liver injury
Latha et al. [70]	2017	India	GSH	250 mg/kg/day orally, 1 week (A)	8	230	14.14	8	95	28.28	8	181	14.14	Stevioside	Liver	Acute Liver injury
Perumal et al. [67]	2016	India	GSH	100 mg/kg/day orally, 3 weeks (A)	6	43.4	7.20	6	11.83	8.23	6	21.4	6.74	Hydroalcoholic	Liver	Diabetes mellitus
Perumal et al. [67]	2016	India	GSH	100 mg/kg/day orally, 3 weeks (A)	6	41.4	9.48	6	19.04	5.07	6	27.43	8.01	Hydroalcoholic	Kidney	Diabetes mellitus
Shivanna et al. [71]	2012	India	GSH	NR orally, 4 weeks (P)	10	24.58	0.51	10	13.58	0.4	10	21.11	0.51	Fraction methanol	Plasma	Diabetes mellitus
Myint et al. [73]	2020	China	GSH	12 mg/kg/day orally, 6 weeks (A)	6	56.75	1.27	6	35.78	1.26	6	36.6	1.24	Rebaudioside A	Liver	Diabetes mellitus
Myint et al. [73]	2020	China	GSH	10 mg/kg/day orally, 6 weeks (A)	6	56.75	1.27	6	35.78	1.26	6	39.89	1.24	Stevioside	Liver	Diabetes mellitus
Singh et al. [37]	2013	India	GSH	300 mg/kg/day orally, 3 weeks(A)	7	25.2	10.05	7	7.1	6.09	7	28.1	19.31	Organic	Liver	Diabetes mellitus
Singh et al. [37]	2013	India	GSH	300 mg/kg/day orally, 3 weeks (A)	7	7.5	6.09	7	4.1	1.32	7	22.1	10.05	Organic	Pancreas	Diabetes mellitus
Singh et al. [37]	2013	India	GSH	300 mg/kg/day orally, 3 weeks (A)	7	22.4	8.73	7	3.5	0.26	7	18.1	8.73	Organic	Kidney	Diabetes mellitus
El-Hadary et al. [60]	2021	Egypt	GSH	300 mg/kg/day orally, 8 weeks (A)	10	80.8	0.9	10	57.2	1.7	10	81.6	1.9	Hydroalcoholic	Liver	Diabetes mellitus
Abdallah et al. [55]	2022	Egypt	GSH	500 mg/kg/day orally, 1 week (A)	7	50	1	7	42.5	2.5	7	48	1.5	Organic	Liver	Liver disease
Abdallah et al. [55]	2022	Egypt	GSH	250 mg/kg/day orally, 1 week (A)	7	50	1	7	42.5	2.5	7	51	2	Stevioside	Liver	Liver disease
Deenadayalan et al. [59]	2021	India	GSH	20 mg/kg/day orally, 45 days (A)	6	13.25	2.45	6	6	2.21	6	9	1.22	Stevioside	Skeletal muscles	Diabetes mellitus
Ranjbar et al. [40]	2020	Iran	TAC	400 mg/kg/day orally, 14 weeks (A)	10	0.36	0.16	10	0.19	0.19	10	0.28	0.13	Hydroalcoholic	Serum	Metabolic syndrome
El Nashar et al. [41]	2022	Egypt	TAC	200 mg/kg/day orally, 4 weeks (P and A)	10	4.65	1.28	10	1.91	1.27	10	6.3	2.63	Organic	Brain	Epilepsy
Abdel-Aal et al. [56]	2021	Egypt	MDA	400 mg/kg/day orally, 3 weeks (A)	8	0.2	0.06	8	0.58	0.06	8	0.24	0.04	Aqueous	Liver	Diabetes mellitus
Abdel-Aal et al. [56]	2021	Egypt	MDA	400 mg/kg/day orally, 3 weeks (A)	8	0.21	0.02	8	0.46	0.03	8	0.24	0.21	Aqueous	Kidney	Diabetes mellitus
Ranjbar et al. [40]	2020	Iran	MDA	400 mg/kg/day orally, 14 weeks (A)	10	34	17.39	10	45	23.72	10	38	12.33	Hydroalcoholic	Serum	Metabolic syndrome
Hussein et al. [62]	2020	Egypt	MDA	400 mg/kg/day orally, 4 weeks (A)	8	1.49	0.03	8	9.9	0.26	8	2	0.13	Hydroalcoholic	Heart	Diabetes mellitus
Mehmood et al. [64]	2019	China	MDA	400 mg/kg/day orally, 8 weeks(A)	8	1.4	0.1	8	5.75	0.9	8	1.75	0.49	Hydroalcoholic	Duodenum	Hyperuricemia
Mehmood et al. [64]	2019	China	MDA	400 mg/kg/day orally, 8 weeks (A)	8	1.2	0.25	8	4.4	0.79	8	1.2	0.95	Hydroalcoholic	Jejunum	Hyperuricemia
Mehmood et al. [64]	2019	China	MDA	400 mg/kg/day orally, 8 weeks (A)	8	1.1	0.37	8	4.1	1.21	8	1.5	0.49	Hydroalcoholic	Ileum	Hyperuricemia
Mehmood et al. [63]	2020	China	MDA	200 mg/kg/day orally, 4 weeks (A)	8	4.6	1.95	8	9.15	1	8	5.75	1.25	Hydroalcoholic	Serum	Hyperuricemia
Elsaid et al. [61]	2019	Egypt	MDA	200 mg/kg/day orally, 5 weeks (A)	12	1.89	0.31	12	5.2	1.07	12	2.92	0.16	Hydroalcoholic	Kidney	Renal ischemia/reperfusion
Ramos-Tovar et al. [43]	2019	Mexico	MDA	100 mg/kg/day orally, 12 weeks (A)	8	0.19	0.03	8	0.29	0.06	8	0.2	0.04	Aqueous	Liver	Liver cirrhosis
Casas-Grajales et al. [58]	2019	Mexico	MDA	20 mg/kg/twice daily intraperitoneally, 8 weeks (A)	8	0.2	0.03	8	0.67	0.09	8	0.4	0.07	Stevioside	Liver	Liver fibrosis
Casas-Grajales et al. [57]	2019	Mexico	MDA	20 mg/kg twice daily intraperitoneally, 8 weeks (A)	8	0.22	0.06	8	0.67	0.11	8	0.32	0.09	Rebaudioside A	Liver	Liver fibrosis
El-Mesallamy et al. [45]	2018	Egypt	MDA	200 mg/kg/day orally, 4 weeks (A)	10	4	1.1	10	11.5	0.9	10	6	0.95	Hydroalcoholic	Skeletal muscles	Diabetes mellitus
El-Mesallamy et al. [45]	2018	Egypt	MDA	2 mg/kg/day orally, 4 weeks (A)	10	4	1.1	10	11.5	0.9	10	8	0.95	Stevioside	Skeletal muscles	Diabetes mellitus
Ramos-Tovar et al. [42]	2018	Mexico	MDA	100 mg/kg/day orally, 10 weeks (A)	8	0.17	0.03	8	0.34	0.09	8	0.24	0.11	Aqueous	Liver	Liver cirrhosis
Ramos-Tovar et al. [68]	2018	Mexico	MDA	100 mg/kg/day orally, 1 week (A)	8	0.10	0.03	8	0.31	0.03	8	0.15	0.06	Aqueous	Liver	Liver cirrhosis
Latha et al. [70]	2017	India	MDA	500 mg/kg/day orally, 1 week (A)	8	35	2.83	8	160	19.8	8	85	5.66	Hydroalcoholic	Liver	Acute liver injury
Latha et al. [70]	2017	India	MDA	250 mg/kg/day orally, 1 week (A)	8	35	2.83	8	160	19.78	8	115	2.83	Stevioside	Liver	Acute liver injury
Moselhy et al. [66]	2016	Saudi Arabia	MDA	200 mg/kg/day orally, 2 weeks (A)	10	5.11	0.14	10	10.14	0.37	10	6.94	0.47	Organic	Liver	Hepatotoxic
Perumal et al. [67]	2016	India	MDA	100 mg/kg/day orally, 3 weeks (A)	6	0.9	0.22	6	2.33	0.56	6	1.06	0.42	Hydroalcoholic	Liver	Diabetes mellitus
Perumal et al. [67]	2016	India	MDA	100 mg/kg/day orally, 3 weeks (A)	6	0.53	0.220	6	2.07	1.13	6	1.49	0.61	Hydroalcoholic	Kidney	Diabetes mellitus
Assaei et al. [44]	2016	Iran	MDA	400 mg/kg/day orally, 4 weeks (A)	10	0.4	0.13	10	1.4	0.25	10	0.45	0.13	Aqueous	Pancreas	Diabetes mellitus
Shivanna et al. [71]	2012	India	MDA	NR, 4 weeks (P)	10	0.06	0.01	10	0.16	0.03	10	0.07	0.01	Fraction methanol	Liver	Diabetes mellitus
Myint et al. [73]	2020	China	MDA	12 mg/kg/day orally, 6 weeks (A)	6	7.12	0.11	6	12.63	0.32	6	12.43	0.23	Rebaudioside A	Liver	Diabetes mellitus
Myint et al. [73]	2020	China	MDA	10 mg/kg/day orally, 6 weeks (A)	6	7.12	0.11	6	12.63	0.32	6	11.89	0.23	Stevioside	Liver	Diabetes mellitus
Singh et al. [37]	2013	India	MDA	300 mg/kg/day orally, 3 weeks (A)	7	25	66.14	7	410	66.14	7	10.2	1.32	Organic	Liver	Diabetes mellitus
Singh et al. [37]	2013	India	MDA	300 mg/kg/day orally, 3 weeks (A)	7	11.1	2.64	7	75.2	47.62	7	4.8	1.32	Organic	Pancreas	Diabetes mellitus
Singh et al. [37]	2013	India	MDA	30 mg/kg/day orally, 3 weeks (A)	7	50.2	31.75	7	415	165.36	7	11	1.32	Organic	Kidney	Diabetes mellitus
El-Hadary et al. [60]	2021	Egypt	MDA	300 mg/kg/day orally, 8 weeks (A)	10	5.33	0.2	10	11.9	0.5	10	4.9	0.1	Hydroalcoholic	Liver	Diabetes mellitus
Morsi et al. [65]	2022	Egypt	MDA	300 mg/kg/day orally, 4 weeks (A)	7	50	1.5	7	160	2	7	76	1.5	Glycosides-sweetener	Ovary	Polycystic ovary syndrome
Abdallah et al. [55]	2022	Egypt	MDA	500 mg/kg/day orally, 1 week (A)	7	4.1	0.5	7	10.9	1.75	7	4.8	0.8	Organic	Liver	Liver disease
Abdallah et al. [55]	2022	Egypt	MDA	250 mg/kg/day orally, 1 week (A)	7	4.1	0.5	7	10.9	1.75	7	5	1	Stevioside	Liver	Liver disease
El Nashar et al. [41]	2022	Egypt	MDA	200 mg/kg/day orally, 4 weeks (P and A)	10	50.76	5.58	10	81.79	5.82	10	42.68	14.6	Organic	Brain	Epilepsy

SOD: superoxide dismutase; CAT: catalase; GPx: glutathione peroxidase; GSH: reduced glutathione; TAC: total antioxidant capacity; MDA: malondialdehyde; A: administration after disease had been established; P: administration prior to disease establishment; NR: not reported, #: number of.

**Table 2:** Results of the multivariate meta-analysis for the assays superoxide dismutase (SOD), catalase activity (CAT), glutathione peroxidase (GPx), reduced glutathione (GSH), total antioxidant activity (TAC), and malondialdehyde (MDA), and stratification according to leaf extract and glycosides. Listed information includes differences between groups with the 95% confidence intervals.

	SOD				CAT				GPx				GSH				TAC			SOD				CAT
Difference	Coef.	*p*-value	95% CI	# Studies	Coef.	*p*-value	95% CI	# Studies	Coef.	*p*-value	95% CI	# Studies	Coef.	*p*-value	95% CI	# Studies	Coef.	*p*-value	95% CI	# Studies	Coef.	*p*-value	95% CI	# Studies
**Overall**																								
control–case	6.09	0.00	3.99, 8.18	21	6.61	0.00	3.24, 9.99	17	31.70	0.05	−0.16, 63.56	4	7.06	0.00	4.46, 9.67	27	1.25	0.00	0.46, 2.03	2	−6.25	0.00	−7.87, −4.63	33
stevia–case	2.73	0.00	1.66, 3.79	21	4.65	0.00	2.02, 7.28	17	25.67	0.03	2.47, 48.88	4	4.92	0.00	2.87, 6.98	27	1.45	0.12	−0.39, 3.27	2	−4.66	0.00	−6.05, −3.27	33
stevia–control	−3.36	0.00	−5.24, −1.48	21	1.96	0.01	−3.51, −0.41	17	−6.03	0.22 **^a^**	−15.62, 3.56	4	−2.14	0.00	−3.33, −0.95	27	0.19	0.79 **^a^**	−1.24, 1.62	2	1.56	0.00	0.72, 2.46	33
**Leaf extract**																								
control–case	4.72	0.00	3.08, 6.37	15	7.66	0.00	3.01, 12.33	13	28.55	0.16	−11.17, 68.28	2	7.67	0.00	3.72, 11.61	20	1.25	0.00	0.46, 2.03	2	−5.49	0.00	−7.15, 0.83	25
stevia–case	2.77	0.00	1.39, 4.15	15	5.73	0.00	1.97, 9.5	13	26.54	0.08	−2.99, 56.06	2	5.89	0.00	2.79, 8.99	20	1.45	0.12	−0.39, 3.27	2	−4.78	0.00	−6.37, −3.19	25
stevia–control	−1.95	0.00	−3.18, −0.72	15	−1.93	0.08 **^a^**	−4.07, 0.19	13	−2.02	0.70 **^a^**	−12.30, 8.27	2	−1.77	0.00	−2.92, −0.62	20	0.19	0.79 **^a^**	−1.24, 1.62	2	0.71	0.00	0.24, 1.17	25
**Glycosides**																								
control–case	11.04	0.00	3.83, 18.25	6	4.49	0.06	−0.09, 9.08	4	36.52	0.29	−31.09, 104.13	2	7.42	0.00	3.41, 11.43	7					−10.28	0.00	−15.02, −5.55	8
stevia–case	2.67	0.00	1.54, 3.81	6	2.43	0.05	0.03, 4.83	4	26.13	0.30	−23.34, 75.60	2	2.99	0.00	1.84, 4.13	7					−3.51	0.00	−4.53, −2.49	8
stevia–control	−8.36	0.01	−15.34, −1.39	6	−2.06	0.11 **^a^**	−4.58, 0.45	4	−10.39	0.26 **^a^**	−28.55, 7.77	2	−4.43	0.06 **^a^**	−9.07, 0.21	7					6.77	0.01	1.27, 12.27	8

*p*-values with **^a^** represent non-statistically significant differences between ‘stevia’ and ‘control’ groups; #: number of.

**Table 3:** Results of the multivariate meta-analysis for the assays superoxide dismutase (SOD), catalase activity (CAT), reduced glutathione (GSH), and total antioxidant capacity (TAC) together and separated according to leaf extract, and stratification according to different types of extract. Listed information includes differences between groups with the 95% confidence intervals.

	SOD, CAT, GSH, TAC				SOD				CAT				GSH				TAC			
Difference	Coef.	*p*-value	95% CI	# Studies	Coef.	*p*-value	95% CI	# Studies	Coef.	*p*-value	95% CI	# Studies	Coef.	*p*-value	95% CI	# Studies	Coef.	*p*-value	95% CI	# Studies
**Combined leaf extract**																				
control−case	6.13	0.00	4.42, 7.84	50	4.72	0.00	3.08, 6.37	15	7.66	0.00	3.01, 12.33	13	7.67	0.00	3.72, 11.61	20	1.25	0.00	0.46, 2.03	2
stevia–case	4.39	0.00	3.03, 5.74	50	2.77	0.00	1.39, 4.15	15	5.73	0.00	1.97, 9.5	13	5.85	0.00	2.79, 8.99	20	1.48	0.12	−0.39, 3.27	2
stevia–-control	−1.74	0.00	−2.46, −1.02	50	−1.95	0.00	−3.18, −0.72	15	−1.93	0.07 **^a^**	−4.07, 0.19	13	−1.77	0.00	−2.92, −0.62	20	0.19	0.79 **^a^**	−1.24, 1.62	2
**Aqueous**																				
control–case	15.66	0.00	3.92, 27.40	9					22.47	0.26	−17.18, 62.12	2	13.53	0.09	−2.03, 29.07	6				
stevia–case	10.79	0.01	2.17, 19.40	9					15.70	0.22	−9.34, 40.75	2	9.78	0.11	−2.34, 21.91	6				
stevia–control	−4.87	0.00	−8.34, −1.41	9					−6.76	0.36 **^a^**	−21.39, 7.86	2	−3.74	0.04	−7.30, −0.17	6				
**Organic**																				
control–case	2.73	0.00	1.49, 3.98	13	2.45	0.00	1.18, 3.73	5	5.18	0.06	−0.35, 10.71	3	2.08	0.01	0.59, 3.55	4				
stevia–case	1.85	0.00	0.77, 2.94	13	0.33	0.65	−1.11, 1.78	5	4.09	0.03	0.29, 7.88	3	2.22	0.00	1.57, 2.87	4				
stevia–control	−0.88	0.13 **^a^**	−2.03, 0.27	13	−2.12	0.08 **^a^**	−4.52, 0.28	5	−1.09	0.29 **^a^**	−3.15, 0.97	3	0.14	0.83 **^a^**	−1.16, 1.45	4				
**Hydroalcoholic**																				
control–case	5.49	0.00	4.20, 6.76	26	4.75	0.00	4.17, 5.33	9	6.58	0.00	3.03, 10.12	7	6.38	0.00	3.35, 9.39	9				
stevia–case	4.26	0.00	2.64, 5.87	26	3.44	0.00	2.28, 4.60	9	5.48	0.06	−0.25, 11.21	7	5.29	0.00	1.83, 8.72	9				
stevia–control	−1.23	0.00	−2.03, −0.43	26	−1.31	0.03	−2.49, −0.11	9	−1.09	0.49 **^a^**	−4.19, 1.99	7	−1.09	0.01	−1.92, −0.27	9				

*p*-values with **^a^** represent non-statistically significant differences between ‘stevia’ and ‘control’ groups; #: number of.

**Table 4:** Results of the multivariate meta-analysis for the assays superoxide dismutase (SOD), catalase activity (CAT), reduced glutathione (GSH), and total antioxidant capacity (TAC) together, and malondialdehyde (MDA) and glutathione peroxidase (GPx) separated according to disease, and stratification according to different types of disease. Listed information includes differences between groups with the 95% confidence intervals.

	SOD, CAT, GSH, TAC				SOD				CAT				GSH				TAC			
Difference	Coef.	*p*- value	95% CI	# Studies	Coef.	*p*- value	95% CI	# Studies	Coef.	*p*- value	95% CI	# Studies	Coef.	*p*- value	95% CI	# Studies	Coef.	*p*- value	95% CI	# Studies
**Overall**																				
control–case	6.13	0.00	4.42, 7.84	50	4.72	0.00	3.08, 6.37	15	7.67	0.00	3.01, 12.33	13	7.67	0.00	3.72, 11.61	20	1.24	0.00	0.46, 2.03	2
stevia–case	4.39	0.00	3.03, 5.74	50	2.77	0.00	1.39, 4.15	15	5.73	0.00	1.97, 9.5	13	5.89	0.00	2.79, 8.99	20	1.44	0.12	−0.39, 3.27	2
stevia–control	−1.74	0.00	−2.46, −1.02	50	−1.95	0.00	−3.18, −0.72	15	−1.93	0.07 **^a^**	−4.07, 0.19	13	−1.77	0.00	−2.92, −0.62	20	0.19	0.79 **^a^**	−1.24, 1.62	2
**Diabetes mellitus**																				
control–case	6.85	0.00	4.92, 8.80	34	3.41	0.00	2.28, 4.54	8	6.35	0.00	2.28, 10.43	6	8.04	0.00	3.70, 12.37	10				
stevia–case	3.75	0.00	2.22, 5.29	34	1.67	0.07	−0.16, 3.49	8	6.22	0.07	−0.43, 12.86	6	6.31	0.00	2.69, 9.92	10				
stevia–control	−3.1	0.00	−4.73, −1.48	34	−1.74	0.08 **^a^**	−3.67, 0.19	8	−0.14	0.94 **^a^**	−3.46, 3.19	6	−1.73	0.06 **^a^**	−3.51, 0.05	10				
**Liver injury**																				
control–case	3.65	0.00	2.76, 4.55	17	3.31	0.00	1.25, 5.37	2	5.49	0.04	0.35, 10.63	3	2.77	0.00	2.02, 3.52	5				
stevia–case	2.74	0.00	1.98, 3.49	17	2.13	0.04	0.08, 4.17	2	3.82	0.08	−0.44, 8.09	3	2.14	0.00	1.48, 2.79	5				
stevia–control	−0.92	0.00	−1.3, −0.54	17	−1.18	0.00	−1.88, −0.48	2	−1.67	0.00	−2.72, −0.62	3	−0.63	0.01	−1.09, −0.17	5				
**Renal disorder**																				
control–case	5.73	0.00	4.43, 7.03	10	5.53	0.00	4.05, 7.02	4	6.96	0.00	5.31, 8.61	2	5.65	0.00	2.52, 8.77	4				
stevia–case	3.80	0.00	2.63, 4.96	10	3.97	0.00	2.21, 5.73	4	3.15	0.01	0.69, 5.61	2	4.06	0.00	1.72, 6.39	4				
stevia–control	−1.93	0.00	−2.66, −1.19	10	−1.56	0.00	−2.43, −0.70	4	−3.82	0.02	−7.07, −0.55	2	−1.59	0.00	−2.51, −0.66	4				

*p*-values with **^a^** represent non-statistically significant differences between ‘stevia’ and ‘control’ groups; #: number of.

**Table 5:** Results of the multivariate meta-analysis for the assays superoxide dismutase (SOD), catalase activity (CAT), reduced glutathione (GSH), malondialdehyde (MDA), and total antioxidant capacity (TAC)) together and separated, malondialdehyde (MDA) and glutathione peroxidase (GPx), and stratification according to different types of tissues. Listed information includes differences between groups with the 95% confidence intervals.

	SOD, CAT, GSH, TAC				SOD				CAT				GSH				TAC			
Difference	Coef	*p*-value	95% CI	# Studies	Coef	*p*-value	95% CI	# Studies	Coef.	*p*-value	95% CI	# Studies	Coef.	*p*-value	95% CI	# Studies	Coef.	*p*-value	95% CI	# Studies
**Overall**																				
control–case	6.13	0.00	4.42, 7.84	50	4.72	0.00	3.08, 6.37	15	7.67	0.00	3.01, 12.33	13	7.67	0.00	3.72, 11.61	20	1.24	0.00	0.46, 2.03	2
stevia–case	4.39	0.00	3.03, 5.74	50	2.77	0.00	1.39, 4.15	15	5.73	0.00	1.97, 9.5	13	5.89	0.00	2.79, 8.99	20	1.44	0.12	−0.39, 3.27	2
stevia–control	−1.74	0.00	−2.46, −1.02	50	−1.95	0.00	−3.18, −0.72	15	−1.93	0.07 **^a^**	−4.07, 0.19	13	−1.77	0.00	−2.92, −0.62	20	0.19	0.79 **^a^**	−1.24, 1.62	2
**Liver**																				
control–case	−5.34	0.00	3.73, 6.95	31	2.93	0.00	1.59, 4.28	6	4.73	0.00	1.85, 7.61	5	4.56	0.00	2.04, 7.07	9				
stevia–case	2.90	0.00	2.12, 3.67	31	2.62	0.00	0.79, 4.45	6	3.04	0.01	0.61, 5.47	5	3.49	0.01	0.97, 6.00	9				
stevia–control	−2.45	0.00	−3.99, −0.9	31	−0.32	0.67 **^a^**	−1.79, 1.16	6	−1.69	0.00	−2.34, −1.04	5	−1.07	0.04	−2.09, −0.05	9				
**Kidney**																				
control–case	5.38	0.00	2.96, 7.79	8	3.85	0.00	2.13, 5.57	2	4.85	0.01	1.36, 8.34	2	6.72	0.01	1.84, 11.60	4				
stevia–case	2.78	0.01	0.58, 4.99	8	−0.12	0.94	−3.28, 3.05	2	2.44	0.20	−1.33, 6.21	2	4.53	0.01	1.00, 8.05	4				
stevia–control	−2.60	0.00	−3.46, −1.73	8	−3.96	0.00	−6.12, −1.81	2	−2.41	0.00	−3.35, −1.48	2	−2.19	0.00	−3.67, −0.71	4				
**Intestine** (duodenum–Jejunum–ileum)																				
control–case	4.96	0.00	3.36, 6.56	6									4.28	0.00	1.96, 6.61	3				
stevia–case	3.44	0.00	1.96, 4.91	6									3.08	0.00	1.18, 4.98	3				
stevia–control	−1.52	0.00	−2.00, −1.05	6									−1.20	0.00	−1.92, −0.48	3				

*p*-values with **^a^** represent non-significant differences between ‘stevia’ and ‘control’ groups; #: number.

**Table 6:** Results of the multivariate meta-analysis for the assay of malondialdehyde (MDA) and stratification according to different types of extract, disease, and tissue. Listed information includes differences between groups with the 95% confidence intervals.

	Difference	Coef.	*p*-Value	95% CI	# of Studies
**Leaf Extract**	control–case	−5.49	0.00	−7.15, 0.83	25
	stevia–case	−4.78	0.00	−6.37, −3.19	25
	stevia–control	0.71	0.00	0.24, 1.17	25
**Aqueous**	control–case	−3.85	0.00	−5.57, −2.14	8
	stevia–case	−3.3	0.00	−4.94, −1.65	8
	stevia–control	0.56	0.01	0.15, 0.96	8
**Organic**	control–case	−5.68	0.00	−8.97, −2.39	6
	stevia–case	−5.06	0.00	−6.82, −3.30	6
	stevia–control	0.62	0.48 ^a^	−1.10, 2.34	6
**Hydroalcoholic**	control–case	−8.33	0.00	−14.08, −2.59	6
	stevia–case	−7.35	0.01	−13.01, −1.69	6
	stevia–control	0.98	0.01	0.25, 1.71	6
**Diabetes melittus**	control–case	−7.33	0.00	−12.25, 2.42	13
	stevia–case	−6.93	0.00	−11.73, −2.12	13
	stevia–control	0.41	0.12 ^a^	−0.10, 0.92	13
**Liver injury**	control–case	−6.33	0.00	−10.04, −2.61	6
	stevia–case	−4.37	0.00	−6.61, −2.13	6
	stevia–control	1.95	0.02	0.35, 3.55	6
**Renal disorder**	control–case	−4.53	0.00	−5.77, −3.29	5
	stevia–case	−3.82	0.00	−5.11, −2.53	5
	stevia–control	0.71	0.01	0.17, 1.25	5
**Blood**	control–case	−1.81	0.15	−4.30, 0.67	2
	stevia–case	−1.32	0.19	−3.29, 0.64	2
	stevia–control	0.49	0.26 ^a^	−0.36, 1.35	2
**Liver**	control–case	−6.96	0.00	−9.72, −4.19	11
	stevia–case	−5.91	0.00	−8.66, −3.16	11
	stevia–control	1.05	0.04	0.04, 2.05	11
**Kidney**	control–case	−3.13	0.00	−4.59, −1.66	4
	stevia–case	−2.45	0.00	−3.91, −0.99	4
	stevia–control	0.68	0.13 ^a^	−0.21, 1.57	4
**Intestine (duodenum–jejunum–ileum)**	control–case	−4.99	0.00	−7.00, −2.98	3
	stevia–case	−4.64	0.00	−6.54, −2.74	3
	stevia–control	0.35	0.23 ^a^	−0.23, 0.93	3
**Pancreas**	control–case	−3.91	0.02	−7.11, −0.71	2
	stevia–case	−3.89	0.01	−6.64, −1.13	2
	stevia–control	0.02	0.95 ^a^	−0.81, 0.85	2

*p*-values with **^a^** represent non-statistically significant differences in MDA assay results; #: number of.

## Data Availability

All data are within the manuscript.
